# Osthole: A Review on Its Bioactivities, Pharmacological Properties, and Potential as Alternative Medicine

**DOI:** 10.1155/2015/919616

**Published:** 2015-07-13

**Authors:** Zhong-Rong Zhang, Wing Nang Leung, Ho Yee Cheung, Chun Wai Chan

**Affiliations:** School of Chinese Medicine, The Chinese University of Hong Kong, Li Wai Chun Building G08, Shatin, New Territories, Hong Kong

## Abstract

This paper reviews the latest understanding of biological and pharmacological properties of osthole (7-methoxy-8-(3-methyl-2-butenyl)-2H-1-benzopyran-2-one), a natural product found in several medicinal plants such as *Cnidium monnieri* and *Angelica pubescens*. *In vitro* and *in vivo* experimental results have revealed that osthole demonstrates multiple pharmacological actions including neuroprotective, osteogenic, immunomodulatory, anticancer, hepatoprotective, cardiovascular protective, and antimicrobial activities. In addition, pharmacokinetic studies showed osthole uptake and utilization are fast and efficient in body. Moreover, the mechanisms of multiple pharmacological activities of osthole are very likely related to the modulatory effect on cyclic adenosine monophosphate (cAMP) and cyclic adenosine monophosphate (cGMP) level, though some mechanisms remain unclear. This review aims to summarize the pharmacological properties of osthole and give an overview of the underlying mechanisms, which showcase its potential as a multitarget alternative medicine.

## 1. Introduction

**Figure 1:**
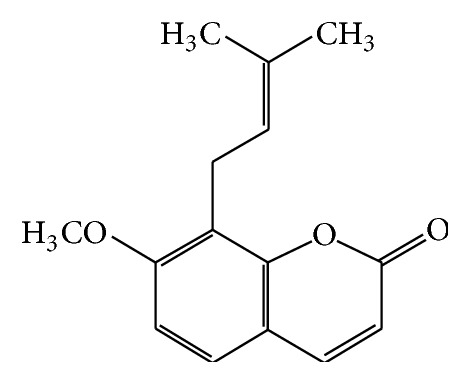
Chemical structure of osthole, the principle component of* Cnidium monnieri*.

**Figure 2:**
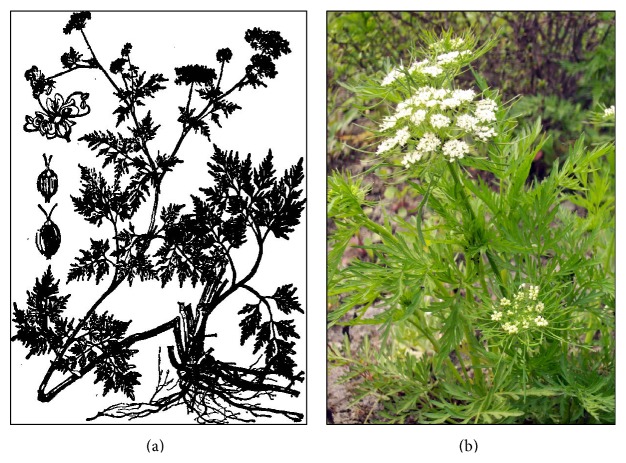
Illustration of the whole plant* Cnidium monnieri* with the fruit structure (a) and photo of the upper ground parts of the herb (b) (modified from http://www.google.com/).

Osthole (also known as osthol), 7-methoxy-8-(3-methyl-2-butenyl)-2H-1-benzopyran-2-one, is a natural coumarin first derived from* Cnidium* plant (Figure 1). High content of osthole is found in the mature fruit of* Cnidium monnieri* (Fructus Cnidii), which is commonly applied in clinical practice of Traditional Chinese Medicine (TCM) (Figure 2), while it is also widely found in other medicinal plants including* Angelica*,* Archangelica*,* Citrus*,* Clausena*. Fructus Cnidii strengthens immune system and improves male function, relieving rheumatic pain and eliminating dampness; most of these medicinal properties are considered to attribute to one of its major bioactive components, osthole [1, 2]. Modern researches have suggested that osthole exhibits antioxidant, anticancer, anti-inflammatory, and immunomodulatory properties [1, 3, 4]. With multiple bioactivities of osthole reported, developing osthole and derivatives as potential multitarget drug should be encouraged. Therefore, it is meaningful to summarize the pharmacological and biological researches on this coumarin, to review the mechanisms behind the effects and get a comprehensive picture of its miscellaneous functions.

## 2. Biological and Pharmacological Activities of Osthole

### 2.1. Nootropic and Neuroprotective Effect

The benefits of osthole and Fructus Cnidii (FC) extract on nervous system have been investigated in recent years. Osthole regulates ion channels and G protein-coupled receptor (GPCR) activities influencing neuronal and neuroendocrine function. Evidence suggested that osthole blocked L-type Ca^2+^ channel and Na^+^ channels in mouse neuronal cells [5, 6]. Osthole increased the affinity of thyrotropin-releasing hormone (TRH) receptor (one of GPCR), hence decreasing the binding of TRH to its receptor and suppressing TRH-evoked production of triphosphoinositol (IP_3_) and mobilization of sequestered Ca^2+^ in rat pituitary GH_4_C_1_ cells [7]. In addition, Wang et al. examined the effect of osthole and imperatorin (another coumarin isolated from FC) on glutamate release from rat hippocampal synaptosomes. The results suggested that both chemicals facilitated 4-aminopyridine- (4-AP-) evoked glutamate release by activating N-and P/Q-type Ca^2+^ channel through a signaling cascade involving protein kinase C (PKC) [8]. Lin et al. then suggested osthole-facilitated glutamate release was related to increased synaptic vesicle availability for exocytosis [9] and to activation of cGMP/PKG-dependent pathway [10]. Osthole was also found to reduce acid-sensing ion channel 3 (ASIC3) expression in rat dorsal root ganglion, which may contribute to its relieving chronic pain from lumbar disc herniation [11]. Moreover, Luszczki et al. reported that osthole showed anticonvulsant effect in maximal electroshock seizure models, suggesting its potential in seizure treatment [12, 13]. Osthole was identified as a modulator of the neurotransmitter gamma-aminobutyric acid (GABA)_A_ receptor* in vitro*, which provided a possible mechanism explaining such antiseizure effect [14, 15].

On the other hand, osthole exhibited neuroprotective effects against neurodegeneration in both* in vitro* and* in vivo* experimental models. Pretreatment of osthole showed significant protective effect on viability of PC12 cells exposed to neurotoxin MPP^+^, an* in vitro* model of Parkinson's disease [16]. Moreover, multiple evidences have demonstrated the protective effect of osthole on alleviating brain damage and improving neurobehavioral functions caused by both chronic [17] and acute [18–20] ischemia due to its antioxidative and anti-inflammatory properties, through mitogen-activated protein kinase (MAPK) pathway by prolonged activation of ERK1/2 and suppression of JNK activity [20]. Osthole has also been suggested as a promising herbal component for memory loss therapy [21]. Animal experiments have been conducted in aluminium chloride- (AlCl_3_-) induced acute senile model [22] and scopolamine-induced amnesia model [23], and results from both studies demonstrated ameliorating effect on memory impairment. In addition, studies demonstrated that osthole was also effective in treating traumatic brain injury by significantly reducing neurological deficits, cerebral edema, and hippocampal neuron loss [24], as well as relieving spatial performance deficits in scopolamine- (SCOP-) treated or ovariectomized (OVX) rats [25], and attenuating autoimmune encephalomyelitis in mice [26].

### 2.2. Osteogenic Activity

Bone modeling effect is one of the bioactivities osthole showing most promising therapeutic potential. Plenty of* in vitro* studies have shown that osthole and coumarin extract from FC promoted proliferation and differentiation of osteoblasts [27–30] and suppress formation and activity of osteoclasts [31, 32], hence tipping the balance in favor of bone remodeling and increasing bone density, which makes osthole a potential agent for osteoporosis treatment. Findings from experiments in both ovariectomy and glucocorticoids-induced osteoporosis rat models supported that treatments with osthole and FC coumarin reduced osteoporotic bone loss [33–36] through estrogen-independent pathway, rather than phytoestrogens commonly found in medicinal herb [36]. Kuo et al. studied the mechanism of osthole-mediated cell differentiation in detail with human osteoblast MG-63 and hFOB. The results obtained after application of bone morphogenetic protein- (BMP-) 2 antagonists, p38 inhibitor, and mitogen-activated protein kinase (ERK2), respectively, indicated that BMP-2/p38 pathway was associated with early phase of osthole-induced osteoblast differentiation, whereas ERK2 was involved in later phase of cellular ossification [33]. A recent paper investigated the potential of osthole in treating and preventing bone loss due to estrogen deficiency and studied the underlying mechanism.* In vitro* findings revealed that osthole promoted osteoblast differentiation by activating Wnt/*β*-catenin signaling and subsequently increasing BMP2 expression.* In vivo* periosteal bone-formation assay by local injection of osthole indicated osthole promoted new bone formation; in addition, comparison of bone microarchitecture, histomorphometric parameters, and biomechanical properties in OVX rat treated with or without osthole revealed that treatment with osthole effectively prevented bone loss in OVX rats [37].

### 2.3. Immunomodulation and Anti-Inflammatory Activity

In late 1990s, a German research group first reported osthole's selective inhibitory effect on 5-lipoxygenase (5-LO) and cyclooxygenase- (COX-) 1 [38, 39]. Both enzymes are critical in the process of inflammation, and inhibition of COX is shown to relieve pain and inflammatory symptoms [40], whereas 5-LO is considered a target for pharmaceutical intervention in various kinds of diseases aside from inflammatory diseases, like cardiovascular diseases, cancer, and osteoporosis [41]. In cell culture, osthole suppressed the immune response of lipopolysaccharide-stimulated macrophages by decreasing reactive oxygen species (ROS) release and inhibiting enzymes including inducible nitric oxide synthase (iNOS), mitogen-activated protein kinases (MAPK), and COX-2 [42, 43]. Osthole also suppressed interleukin- (IL-) 4 and tumor necrosis factor- (TNF-) *α* induced eotaxin expression in bronchial epithelial BEAS-2B cells [44] and inhibited hypertrophic scar fibroblasts through inducing apoptosis and downregulating TGF-*β* expression [45]. Similar anti-inflammatory activates of osthole have also been found in rat peritoneal cells and human peripheral blood mononuclear cells [46]. Meanwhile, anti-inflammatory effect of osthole was also confirmed in animal models. Results from carrageenan-induced hind paw edema in rats suggested that osthole suppressed production of prostaglandin (PG), nitric oxide (NO), and malondialdehyde (MDA), as well as downregulated activity of NOS, which was likely to associate with the elevation of cyclic adenosine monophosphate (cAMP) and cyclic adenosine monophosphate (cGMP) levels [47]. Osthole also inhibited the expression of COX-2 and NOS in dorsal root ganglion and relieved mechanical allodynia in rat model of sciatica induced by lumbar disc herniation [48]. The chemical was also reported to have antiallergic effect in passive cutaneous anaphylaxis (PCA), 2, 4-dinitrofluorobenzene (DNFB), and picryl chloride- (PC-) induced contact dermatitis in experimental animals [49].

### 2.4. Anticancer Effect

Accumulating experimental evidences have shown that osthole exhibited antiproliferative properties and induced apoptosis in various kinds of tumor cells [2, 50], including leukemia HL-60 [51], L1210 [52]; prostatic cancer cell LNCaP, PC3, and DU145 [52]; cervical cancer cell Hela [53]; ovarian cancer cell SKOV3 [54]; lung cancer cell A549 [55]; SK-LU-1 [56]; epidermal cancer cell KB [56]; breast cancer cell MCF-7 [54, 56, 57], MDA-MB-231 [54, 57], and 4T1 [57]; and hepatocellular carcinoma (HCC) HepG2 [56], SMMC-7721, and Hepa1-6 [58]. The antitumor activities of osthole have also been supported with* in vivo* results showing prolonged survival days of P-388 D1 tumor-bearing CDF mice [53] and suppressed tumor growth in HCC tumor models established by injection of SMMC-7721 or Hepa1-6 cells [58]. It is noteworthy that osthole successfully inhibited the migration and invasion of metastatic cancer cells. Yang et al. firstly reported that osthole inhibited migration of MCF-7 cells and invasion of MDA-MB-231 cells and suggested that suppression of matrix metalloproteinases (MMP) enzyme activities might be the possible mechanism [57]. Prevention of cell migration and invasion was also reported in human lung adenocarcinoma CL1-5, H1299, and A549 [59]. Additionally, combination of osthole and other nature products showed synergetic effect on inhibition of tumor cell proliferation and invasion [60–62].

The molecular mechanism of anticancer effect of osthole still remains unclear. It is highly likely to be combinatory effects on carcinogenesis and tumor progression. Osthole has been suggested to modulate PI3K/Akt signaling pathway leading to G2/M arrest and apoptosis in lung cancer A549 cells [55]. It downregulates FASN, which is highly expressed in many solid tumors, in HER2-overexpressing breast cancer cells through inhibiting the c-Met/Akt/mTOR pathway [54, 63]. Osthole also normalized plasma alanine aminotransferase (ALT) which has been proved to be a strategy for preventing the development of HCC [64]. Moreover, osthole inhibited cancer cells invasion and transition through suppression of MMP-2 and MMP-9 which were induced by a serial of inflammatory factors [57, 59, 61, 62]. Besides, osthole was demonstrated as promising therapeutic agent for cancer treatment due to histone deacetylase inhibition [65, 66].

### 2.5. Hepatoprotective Effect and Benefits on Metabolic Diseases

Liver is regarded as one of the vital organs that functions as a center for metabolism of nutrients and excretion of wastes. Osthole exerts protective effects against hepatitis. It suppressed the secretion of hepatitis B virus (HBV) in cell culture [67] and prevented hepatitis in mice induced by concanavalin A or anti-Fas antibody [68–70]. Osthole also exhibited therapeutic effect on both hyperlipidemic [71–77] and alcoholic fatty liver animals [71, 75, 78, 79]. Mechanism studies revealed that osthole modulated expression of multiple lipogenic genes [77, 80], increased adiponectin release, and hence improved insulin resistance [76] via activation of PPAR*α*/*γ* pathway. In addition, antifibrotic activity of osthole on HSC-T6 hepatic stellate cell lines was also reported [81].

Osthole is not only beneficial to liver but also beneficial to endocrine system and metabolism of the whole body. Screening for allosteric modulators of melanocortin-4 receptor, a target for obesity and cachexia therapy, reported osthole and other two FC coumarins as potential modulators [82]. Osthole is regarded as a potential antidiabetic agent as well.* In vitro* and* in vivo* experiments demonstrated that osthole alleviated hyperglycemia by activating PPAR*α*/*γ*, AMP-activated protein kinase (AMPK), and downstream acetyl CoA carboxylase [83, 84]. In addition, the Yang tonifying effect of osthole has been explored in modern experimental way. Oral administration of osthole significantly increased androgen, gonadotropin production, and NOS activity in immature castrate male rats [85]. Administration of osthole and FC coumarin extract improved immunological function in kidney yang deficiency animal models induced by hydrocortisone acetate [86], via elevated function of pituitary-thyroid [87], and pituitary-adrenocortex axis [88].

### 2.6. Vasorelaxant Properties and Cardiovascular Benefits

Osthole exhibits protective effect on heart and circulatory system. Abnormal vascular smooth muscle cell proliferation is a major component of vascular disease including atherosclerosis, vein graft occlusion, and restenosis after angioplasty, whereas osthole treatment selectively inhibits the proliferation of those vascular smooth muscle cells [89]. Osthole showed vasorelaxant properties due to its Ca^2+^-channel antagonistic effect and upregulation of cGMP level in vascular smooth muscle [90–92]. Osthole was also proposed to prevent isoprenaline-induced fibrosis through activating of PPAR*α*/*γ* and subsequent suppression of NF-*κ*B production in myocardial tissues [93]. Additionally, accelerative effect on *β*-oxidation of hepatic fatty acids in hypertensive rats suggested it might be useful for prevention of atherosclerosis [80]. Osthole suppresses platelet aggregation via inhibition of thromboxane formation and phosphoinositides breakdown, thus making it a prospective antithrombotic agent [94, 95]. Osthole exerts relaxant effect not only on blood vessels, but also on other tissues such as isolated rodent ileum and taeniae coli [96]; trachea [97]; and corpus cavernosum, which partially explain long history of using FC as a herbal medicine to improve male sexual dysfunction [98, 99].

### 2.7. Antimicrobial and Antiparasitic Effect

Osthole exerts antifungal properties on numbers of fungi species. Experimental evidences showed that osthole inhibited hypha growth* of Fusarium graminearum*, a parasite found on common weeds and cereal crops, through glucose starvation [100], and controlled powdery mildew caused by* Sphaerotheca fuliginea* via inhibiting spore germination and mycelia growth [101]. Osthole derivatives exhibited curative effect on pepper blight caused by* Phytophthora capsici* [102]. Other antibacterial activities on both gram positive and gram negative bacteria were also reported [103, 104]. Osthole was also found to exhibit antiviral activity not only on HBV, but also on HIV-1 by inhibiting Rev-export, which is critical in HIV-1 entails replication [105]. In addition, anthelmintic activity of osthole was also noted in goldfish against* Dactylogyrus intermedius* [106, 107].

## 3. Pharmacokinetics and Metabolism of Osthole

Pharmacokinetics of osthole in rat plasma after oral or intravenous administration was studied using HPLC method, yielding concentration/time curve with rapid distribution followed by a slower elimination phase [108–111]. Intestinal absorption of osthole was studied with HPLC in rat single pass intestine perfusion (SPIP) model, where results showed osthole absorption was a passive diffusion process in whole intestinal sections [112]. Osthole metabolism after oral administration was studied in male SD rats and 10 phase I and 3 phase II metabolites were isolated and identified from urine. The major phase I metabolic reactions were hydroxylation, demethylation, and hydrogenation, while glucuronidation contributed to phase II metabolism [113]. Absorption and metabolism of osthole were also investigated in human colorectal Caco-2 cell model. Osthole demonstrated high absorptive permeability and accumulation in Caco-2 cells; major phase I metabolites were desmethyl-osthol and its multiple isomers [114, 115].

## 4. Discussion

### 4.1. Composite Bioactivities of Osthole on Body System

**Figure 3:**
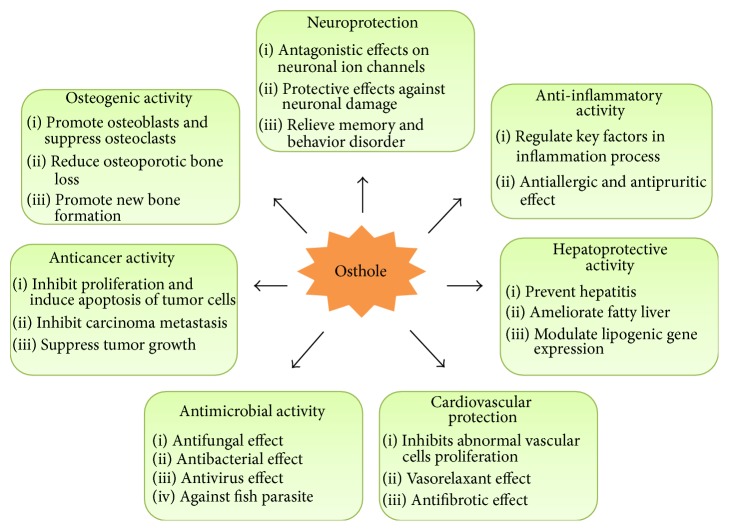
Multiple systemic pharmacological and beneficial effects and related experimental results.

Osthole exerts a broad spectrum of biological and pharmacological activities. While effects of osthole are categorized under different biological activities in this paper, a lot of connections can actually be found among them (Figure 3). Osthole exhibits immunomodulatory and anti-inflammatory properties, by regulating the expression of a series of key factors, including TNF-*α*, NF-*κ*B, TGF-*β*, COX, NO, ERK, and JNK, involved in the process of immune response and other metabolic and biological processes. For instance, NF-*κ*B plays important role in modulating immunological response, and disturbance of NF-*κ*B expression has been linked to some autoimmune diseases, cancer, and many other diseases. Likewise, TGF-*β* is a cytokine involving in several key pathways which are related to the development of numerous diseases. Modulation by osthole on those critical factors probably contributes to its various benefits on systemic level. Principally, in various tissues and organs, the anti-inflammatory and antioxidative activities of osthole induce and magnify its anticancer properties and protective effects on other systems. Similarly, osthole showed antifibrotic effect in both hepatic and myocardial tissues, which contribute to the protective effect on liver and cardiovascular system. Osthole has also been suggested as a natural activator of defective DeltaF508-cystic fibrosis transmembrane conductance regulator (CFTR) chloride channel gating and thus may be a lead compound for cystic fibrosis therapies [116, 117]. In addition, liver plays a critical role in metabolism including catabolism, storage, and anabolism. Hence, the numerous benefits on liver certainly contribute to better metabolic system of human body. Moreover, the positive influence of osthole to male sexual dysfunction has been found related to vasorelaxant activity which was also reported in other tissues such as ileum and taeniae coli and thoracic aorta.

### 4.2. cAMP and cGMP Level

**Figure 4:**
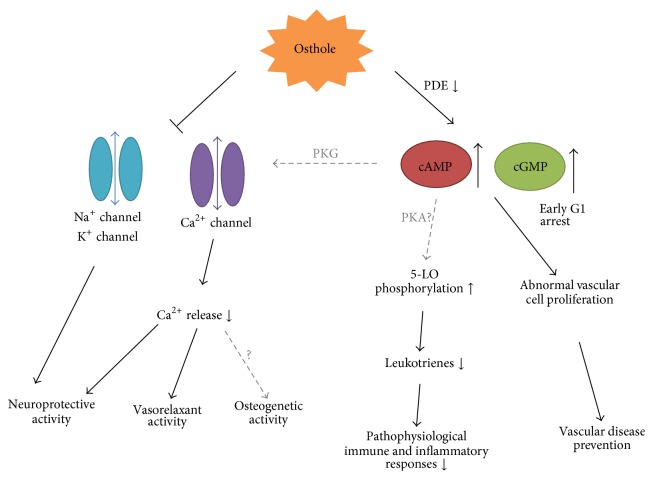
Possible interrelation between effect of osthole on intracellular ion channels, cyclic adenosine monophosphate (cAMP), and cyclic guanosine monophosphate (cGMP) levels with some of its pharmacological activities. (Hypotheses unconfirmed in studies of osthole are indicated with dashed line.)

Accumulating evidence in studies for biological activities of osthole revealed that osthole exerts a nonspecific elevation of intracellular and tissue cAMP and cGMP, which is likely involved in the underlying mechanism of some bioactivities of osthole. cAMP and cGMP, derived from ATP and GTP, respectively, are second messenger prominent in many biological processes. cAMP mainly works by activating protein kinase A (PKA), as well as directly affecting ion channels and growth hormone. cGMP is also a regulator of ion channels related to cell cycle arrest, apoptosis, and smooth muscle tissue relaxing as well. Osthole increases cAMP and cGMP level by inhibiting cAMP and cGMP phosphodiesterases (PDEs) which hydrolyze cAMP and cGMP by degradation of the phosphodiester bond [95, 97]. The inhibitory effect on vascular smooth muscle cell was found to associate with osthole-induced elevation of cAMP and cGMP level [89], whereas osthole-facilitated glutamate release in hippocampal synaptosomes was associated with activation of cGMP/PKG-dependent pathway [10]. Osthole-mediated suppression of inflammatory factors in carrageenan-induced model was noted to attribute to cGMP elevation [47]. Moreover, researchers suggested that vasorelaxant property of osthole was linked to increased cAMP and cGMP levels caused by osthole treatment [92, 97, 99] (Figure 4).

### 4.3. Ion Channel Regulator

Osthole has been found to influence membrane potential and some types of ion channels in various cells and tissues, including sodium channel, acid-sensing ion channel, chloride channel of CFTR, and especially calcium channel. Effect of osthole on these ion channels is closely related to bioactivities of osthole as vasorelaxant, immunomodulatory, antifibrotic effects, and, in particular, its neuronal and neuroendocrine functions including neuroprotective, anticonvulsant, and pain relieving properties. On the other hand, calcium concentration regulates the osteoprogenitors behavior which play important roles in both bone homeostasis and regeneration [117, 118], thereby making calcium channel a prospective direction to study the mechanism of osteogenic effect of osthole. cAMP and cGMP have been well documented as important regulators of L-type Ca^2+^ channel and some other ion channels [119–121]. Therefore, effect of osthole on ion channels is at least partially induced by modulation of cAMP and cGMP level (Figure 4).

### 4.4. 5-Lipoxygenase Inhibitor

5-lipoxygenase (5-LO) is a rate-limiting dioxygenase in the process of leukotrienes (LTs) biosynthesis from the precursor arachidonic acid. As LTs are key mediators in immune and inflammatory responses in pathophysiology of numbers of respiratory and cardiovascular diseases, 5-LO is regarded as a target in developing therapy of related diseases and 5-LO inhibitors are being developed as a treatment approach. Osthole has been reported to be a 5-LO inhibitor in previous studies. Meanwhile, researchers proposed that signaling through cAMP/PKA results in phosphorylation and cytoplasmic sequestration of 5-LO and inhibition of LTs synthesis. It has been found that many cAMP-elevating agents such as isoproterenol, prostaglandin E, and prostaglandins attenuate 5-LO translocation and LTs biosynthesis [122–124]. Therefore, inhibitory effect of osthole on 5-LO may also be associated with osthole-mediated cAMP elevation.

## 5. Conclusion

Plenty of experimental results demonstrated that osthole exhibits a variety of pharmacological benefits including neuroprotection, osteogenesis, immunomodulation, and cancer-combating properties, making it a potential multitarget complementary medicine and functional food. The mechanisms underlying these properties have not been fully understood, yet the regulatory effect of osthole on cAMP and cGMP level and some ion channels can be seen as contributing to several of those properties. Further studies are needed to enrich the knowledge of the pharmacological effects and address the safety issues of osthole in order to develop this natural product and its derivatives as an agent for disease prevention and therapeutics in human.
